# Current Status and Future Perspective of Stenting for Symptomatic Intracranial Atherosclerotic Disease: A Meta-Analysis

**DOI:** 10.1155/2017/3258681

**Published:** 2017-06-18

**Authors:** Zhong-Hao Li, Zhen-Hua Zhou, Xian-Jin Zhu, Wei Liu, Ya-Wen Chen, Zi-Yao Chen, Zun-Jing Liu

**Affiliations:** ^1^Graduate School, Beijing University of Chinese Medicine, Beijing 100029, China; ^2^Department of Neurology, China-Japan Friendship Hospital, Beijing 100029, China; ^3^Department of Neurology, Southwest Hospital, Third Military Medical University, Chongqing 400038, China; ^4^Department of Radiology, China-Japan Friendship Hospital, Beijing 100029, China

## Abstract

The aim of this study was to evaluate the safety and effectiveness of percutaneous transluminal angioplasty and stenting (PTAS) for intracranial atherosclerotic disease (ICAD) by conducting a meta-analysis. Two independent observers searched PubMed, EMBASE, and Cochrane Library for relevant studies up to 31 December 2016. A meta-analysis was conducted using Review Manager 5.3. Three studies involving 581 cases were included. The meta-analysis indicated that any stroke (RR = 3.13; 95% CI: 1.80–5.42), ischemic stroke (RR = 2.15; 95% CI: 1.19–3.89), and intracranial hemorrhage (RR = 14.71; 95% CI: 1.96–110.48) within 30 days in medical therapy alone were lower compared with PTAS plus medical therapy, but there were no significant differences in any stroke and ischemic stroke beyond 30 days between the two groups. There were also no significant differences in any death and myocardial infarction between the two groups. This meta-analysis demonstrated that, compared with medical therapy alone, PTAS for ICAD had a high risk of complication, but most complications in PTAS group occurred within 30 days after the operation, and beyond 30 days the PTAS was not inferior compared with medical therapy alone. Further studies are needed to reduce the periprocedural complications and reappraise the PTAS.

## 1. Introduction

Intracranial atherosclerotic disease (ICAD) is a common cause of stroke and associated with a high risk of recurrent stroke [1, 2]. Its incidence and prevalence vary by ethnicity. ICAD is more common in Asians, Hispanics, and those of African descent, compared to Caucasians. It causes approximately 10% of all strokes in the USA [3, 4]. In Asian studies, ICAD accounts for 33–50% of all strokes in China, 47% in Thailand, 48% in Singapore, and 10–25% in Korea [5]. In National Institute of Health-sponsored, multicenter Warfarin-Aspirin Symptomatic Intracranial Disease (WASID) trial [6], 14% and 23% of the patients with a transient ischemic attack (TIA) or stroke attributable to a high-grade (50–99%) intracranial stenosis had a further ipsilateral ischemic stroke over the next year despite medical therapy. Consequently, alternative therapies are urgently needed for these patients.

Over the past decade, intracranial percutaneous transluminal angioplasty and stenting (PTAS), including the use of balloon-mounted stent or self-expanding stent, has increasingly been used in clinical practice all around the world [7–9]. The first randomized trial, stenting versus aggressive medical therapy for intracranial arterial stenosis (SAMMPRIS) trial, was reported in 2011 [10]. The rate of 30-day stroke or death in PTAS group was 14.7%, which was much higher than expected and implied that aggressive medical management was superior to PTAS. Criticisms regarding the design in SAMMPRIS have been raised including the inexperience of the operators and poor patient selection. Lessons learned from SAMMPRIS changed the design on patient selection, stenting techniques, and periprocedural management [11]. After that several well designed clinical trials were reported, and several trials showed a lower rate of 30-day stroke or death. Against the background of an increasing amount of data on this endovascular therapy field, we systematically searched the relevant studies, which compared the immediate and long term outcomes between PTAS plus medical therapy and medical therapy alone for ICAD.

## 2. Methods

### 2.1. Inclusion Criteria and Exclusion Criteria

Studies were considered for inclusion if they met the following criteria: (1) all published randomized, controlled trials (RCTs) were comparing PTAS plus medical therapy and medical therapy alone and more than 3 patients enrolled in each group; (2) all patients had been treated for an atherosclerotic intracranial stenosis greater than 50% which located in intracranial segment of internal carotid artery, middle cerebral artery, and vertebral or basilar artery; and (3) periprocedural complications were reported. Studies were considered for exclusion if they met the following criteria: (1) follow-up time was less than 1 year and (2) complication rate could not be extracted.

### 2.2. Search Strategy and Data Extraction

Two independent observers searched PubMed, EMBASE, and Cochrane Library for the relevant studies published in English up to 31 December 2016. The key words included “intracranial arteriosclerosis”, “cerebral arteriosclerosis”, “stenosis”, “stent”, and “randomized controlled trial”. Two reviewers independently reviewed the citations, abstracts, and full-text articles and determined the eligibility of all the studies identified in the initial search. When the entire process was completed, the two cross-checked with each other. In cases of disagreements, a third reviewer was consulted. We systematically reviewed any stroke, ischemic stroke, intracranial hemorrhage, any death, myocardial infarction, and so on during follow-up period reported in all the trials. For RCTs, the following details were extracted: participants, follow-up time, eligibility criteria, stenosis rate, stenosis location, and primary end points. Articles that met all inclusion criteria but specific data extraction was not possible were marked as “NG” (not given). After systemic review, data of any stroke, ischemic stroke, intracranial hemorrhage, any death, and myocardial infarction within 30 days and during the follow-up were used in meta-analysis.

### 2.3. Quality Assessment

Assessment of the quality of the included studies was performed using the methodology recommended by Cochrane Handbook for Systematic Reviews of Interventions [12]. This method comprised assessments of the risk of potential bias in seven domains: random sequence generation (low risk, high risk, or unclear risk), allocation concealment (low risk, high risk, or unclear risk), blinding of outcome assessment (low risk, high risk, or unclear risk), blinding of participants and personnel (low risk, high risk, or unclear risk), incomplete outcome data (low risk, high risk, or unclear risk), selective reporting (low risk, high risk, or unclear risk), and other biases (low risk, high risk, or unclear risk), such as the baseline, source of funding, and academic biases. Two reviewers will independently assess the quality of the included trials. Discrepancies will be resolved by mutual consensus with a third author.

## 3. Statistical Analysis

Statistical analysis was performed using Review Manager Version 5.3 software (Cochrane Collaboration, Oxford, UK). We conducted separate meta-analysis according to different groups. The heterogeneity of the qualitative analysis was assessed by Chi-square test, and the significant level was set to *P* = 0.1. We used *I*^2^ to conduct quantitative analysis of heterogeneity. The significant level was set to 50%. If *P* > 0.1 and *I*^2^ < 50%, the different RCTs can be regarded as homogeneous. If *P* < 0.1 and *I*^2^ ≥ 50%, the different RCTs can be regarded as heterogeneity. All pooled effect estimates were assessed using random effects model. We used weighted mean deviation (WMD) and 95% confidence interval (CI) to represent the continuous data, and the dichotomous data can be described by risk ratio (RR) and 95% CI. Our meta-analysis has been registered (URL: https://www.crd.york.ac.uk/PROSPERO/; unique identifier: CRD42015024370).

## 4. Results

**Figure 1:**
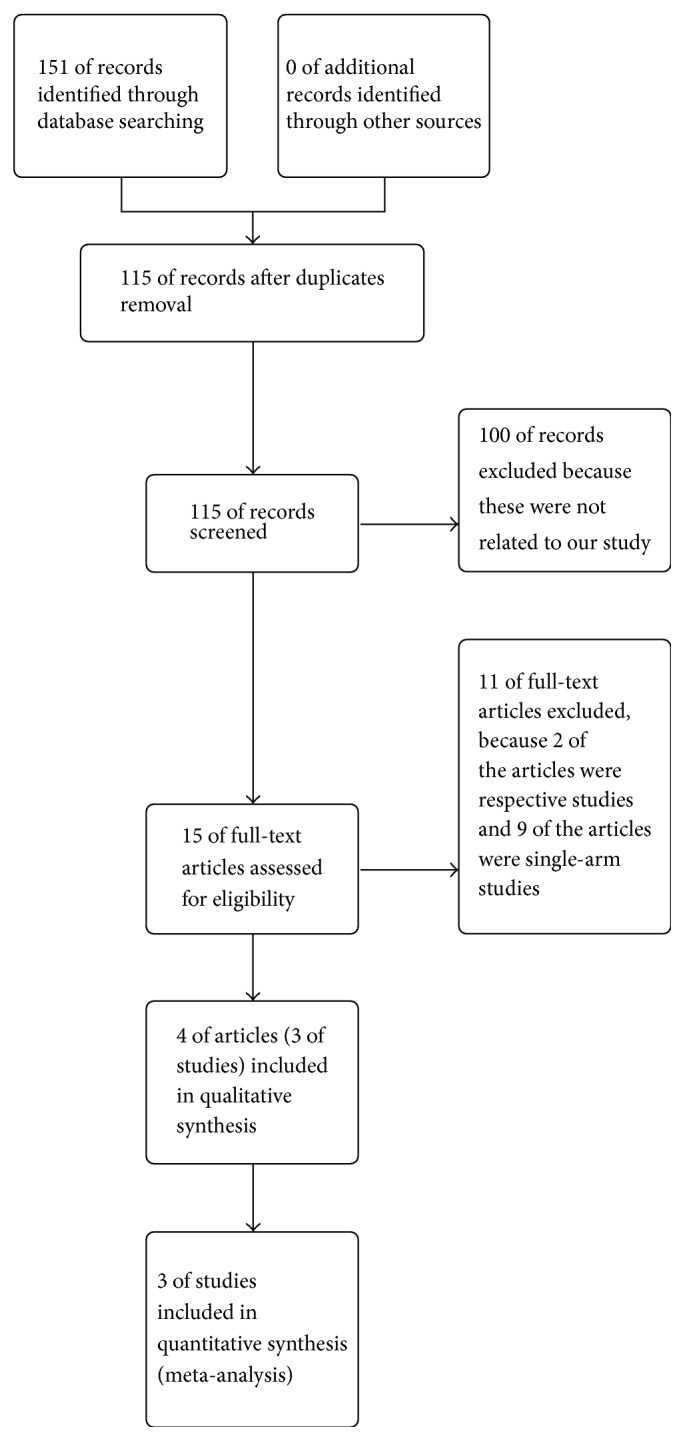
Flow diagram of the controlled trials reviewed for this meta-analysis.

The 151 potentially relevant trials were identified from the databases in the initial search, and then 36 duplicate trials were excluded. The search identified 115 citations. Finally, only 3 studies involving 581 cases met the inclusion criteria (Figure 1). All the 3 studies—the Stenting and Aggressive Medical Management for Preventing Recurrent stroke in Intracranial Stenosis (SAMMPRIS) trial was reported in 2011 [10] and 2014 [13], the Vitesse™ Intracranial Stent Study for Ischemic Stroke Therapy (VISSIT) trial was reported in 2015 [14], and Vertebral Artery Stenting trial (VAST) was reported in 2015 [15]—described participants, follow-up time, eligibility criteria, stenosis rate, stenosis location, and primary end points (Table 1). For SAMMPRIS, with regard to data within 30 days we used the data published in 2011, because this article was written when the last patient enrolled completed the 30-day evaluation, and, with regard to data in 1 year or longer, we used the data published in 2014 because this was the final result of SAMMPRIS trial.

### 4.1. Quality Assessment of the Included RCTs

**Figure 2:**
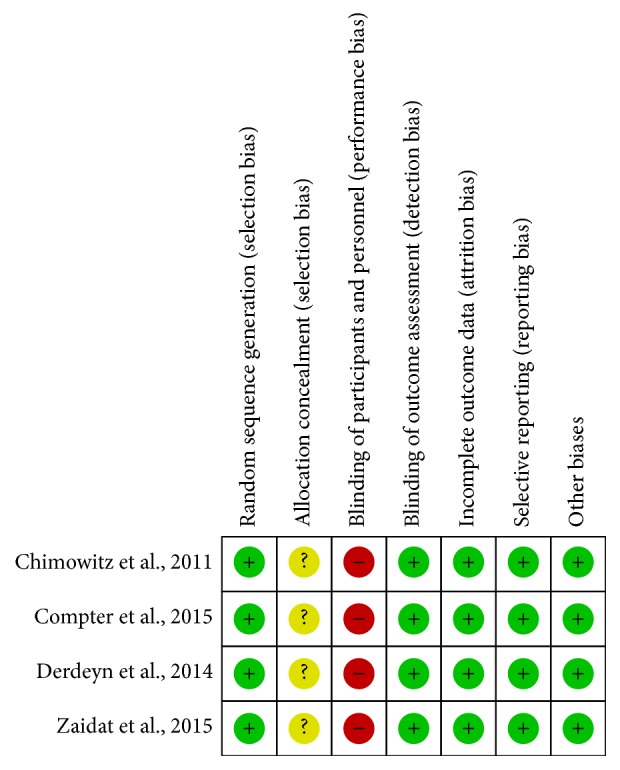
Quality assessment for included RCTs.

All 3 RCTs mentioned “random” and described the method of generating a random sequence. Because only one of the treatment groups underwent stenting, the trial could not be double masked. All of the studies described the case where subjects quit or were lost to follow-up. The number of subjects that quit or were lost to follow-up of each study were less than 20% of the total number. Therefore, we considered the data integrity was good. The detailed assessments are shown in Figure 2.

### 4.2. Any Stroke within 30 Days, beyond 30 Days, between 30 Days and 1 Year, within 1 Year, and during the Follow-Up

**Figure 3:**
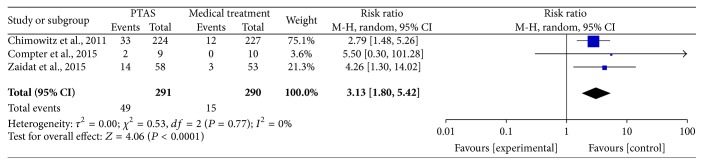
Forest plot of any stroke within 30 days for PTAS plus medical therapy versus medical therapy alone.

**Figure 4:**
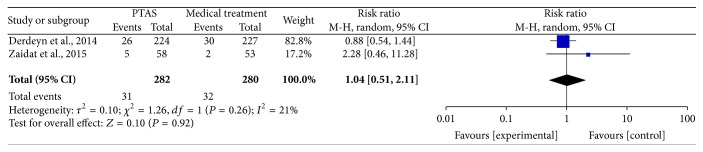
Forest plot of any stroke beyond 30 days for PTAS plus medical therapy versus medical therapy alone.

**Figure 5:**
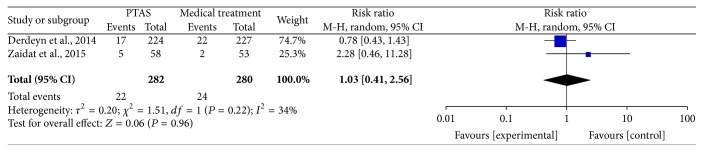
Forest plot of any stroke between 30 days and 1 year for PTAS plus medical therapy versus medical therapy alone.

**Figure 6:**
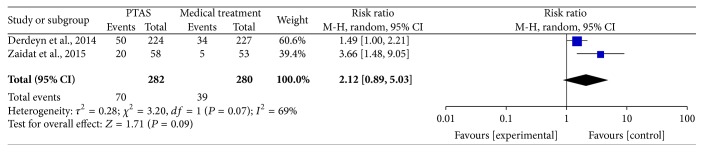
Forest plot of any stroke within 1 year for PTAS plus medical therapy versus medical therapy alone.

**Figure 7:**
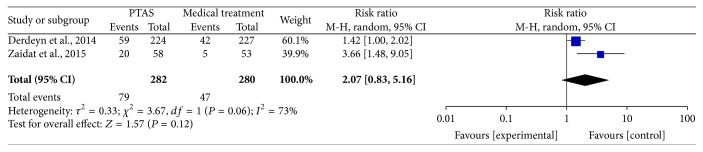
Forest plot of any stroke during the follow-up for PTAS plus medical therapy versus medical therapy alone.

All of the 3 studies reported any stroke (including ischemic stroke and hemorrhage stroke) within 30 days, and the SAMMPRIS and VISSIT trials also reported any stroke within 1 year and during the follow-up. The median duration of follow-up in SAMMPRIS trial was 32.4 months (IQR 24.2–40.5; range: 0 –52.6 months); and the median follow-up time in VISSIT trial was 10.5 months (range: 0–51 months). Comparing PTAS plus medical therapy with medical therapy alone, there was no heterogeneity from any stroke within 30 days (*P* = 0.77; *I*^2^ = 0%), any stroke beyond 30 days (*P* = 0.26; *I*^2^ = 21%), and any stroke between 30 days and 1 year (*P* = 0.22; *I*^2^ = 34%). The pooled results showed significant difference in any stroke within 30 days (RR = 3.13; 95% CI: 1.80–5.42; Figure 3) but had no significant differences in any stroke beyond 30 days (RR = 1.04; 95% CI: 0.51–2.11; Figure 4) and between 30 days and 1 year (RR = 1.03; 95% CI: 0.41–2.56; Figure 5). There was heterogeneity from any stroke within 1 year (*P* = 0.07; *I*^2^ = 69%) and any stroke during the follow-up (*P* = 0.06; *I*^2^ = 73%) between two groups. The pooled results showed significant difference in any stroke within 1 year (RR = 2.12; 95% CI: 0.89–5.03; Figure 6) and any stroke during the follow-up (RR = 2.07; 95% CI: 0.83–5.16; Figure 7).

### 4.3. Ischemic Stroke within 30 Days, beyond 30 Days, and during the Follow-Up

**Figure 8:**
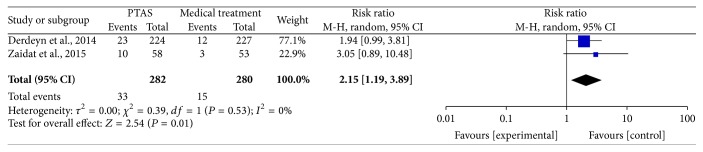
Forest plot of ischemic stroke within 30 days for PTAS plus medical therapy versus medical therapy alone.

**Figure 9:**
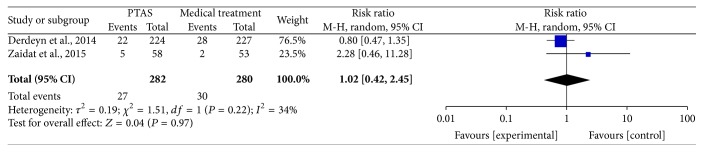
Forest plot of ischemic stroke beyond 30 days for PTAS plus medical therapy versus medical therapy alone.

**Figure 10:**
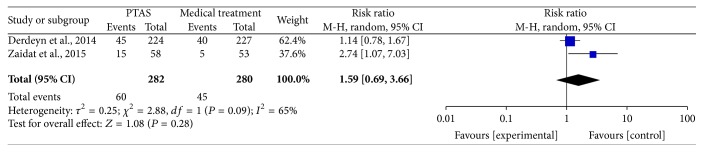
Forest plot of ischemic stroke during the follow-up for PTAS plus medical therapy versus medical therapy alone.

The SAMMPRIS and VISSIT trials reported the ischemic stroke as serious adverse events within 30 days and during the follow-up, including ischemic stroke in the territory of qualifying symptomatic artery and ischemic stroke not in the territory of qualifying symptomatic artery and cerebral infarction. Comparing PTAS plus medical therapy with medical therapy alone, there was no heterogeneity from the ischemic stroke within 30 days (*P* = 0.53; *I*^2^ = 0%) and beyond 30 days (*P* = 0.22; *I*^2^ = 34%). The pooled results showed significant difference in ischemic stroke within 30 days (RR = 2.15; 95% CI: 1.19–3.89; Figure 8) but no significant difference in ischemic stroke beyond 30 days (RR = 1.02 95% CI: 0.42–2.45; Figure 9). There was heterogeneity from the ischemic stroke during the follow-up (*P* = 0.09; *I*^2^ = 65%) between two groups. The pooled results showed no significant difference (RR = 1.59; 95% CI: 0.69–3.66; Figure 10).

### 4.4. Intracranial Hemorrhage within 30 Days and during the Follow-Up

**Figure 11:**
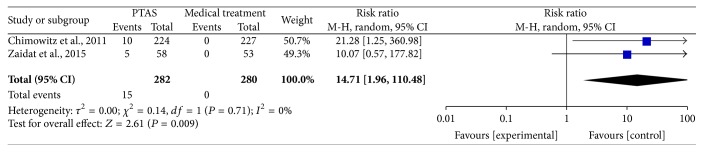
Forest plot of intracranial hemorrhage within 30 days for PTAS plus medical therapy versus medical therapy alone.

**Figure 12:**
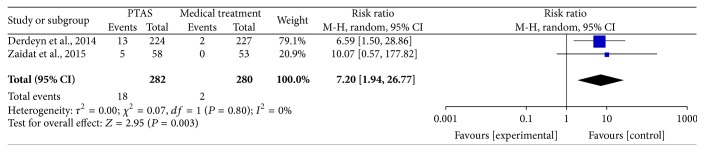
Forest plot of intracranial hemorrhage during follow-up for PTAS plus medical therapy versus medical therapy alone.

The SAMMPRIS and VISSIT trials reported the intracranial hemorrhage as serious adverse events during the follow-up, including intracranial hematoma, symptomatic intracranial hemorrhage, and asymptomatic intracranial hemorrhage. Comparing PTAS plus medical therapy with medical therapy alone, there was no heterogeneity from the intracranial hemorrhage within 30 days (*P* = 0.71; *I*^2^ = 0%) and during the follow-up (*P* = 0.80; *I*^2^ = 0%). The pooled results showed significant differences in intracranial hemorrhage within 30 days (RR = 14.71; 95% CI: 1.96–110.48; Figure 11) and during the follow-up (RR = 7.20; 95% CI: 1.94–26.77; Figure 12).

### 4.5. Any Death within 30 Days, beyond 30 Days, between 30 Days and 1 Year, within 1 Year, and during the Follow-Up

**Figure 13:**
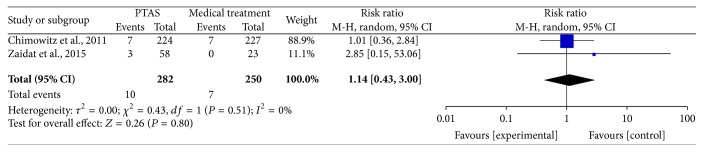
Forest plot of death within 30 days for PTAS plus medical therapy versus medical therapy alone.

**Figure 14:**
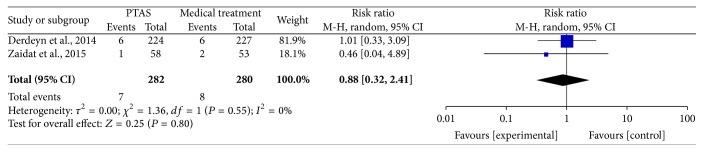
Forest plot of death beyond 30 days for PTAS plus medical therapy versus medical therapy alone.

**Figure 15:**
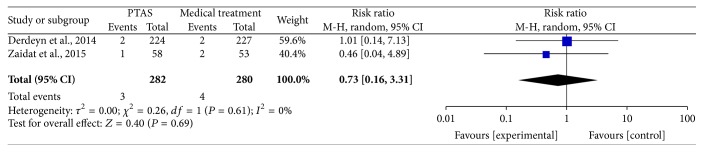
Forest plot of death between 30 days and 1 year for PTAS plus medical therapy versus medical therapy alone.

**Figure 16:**
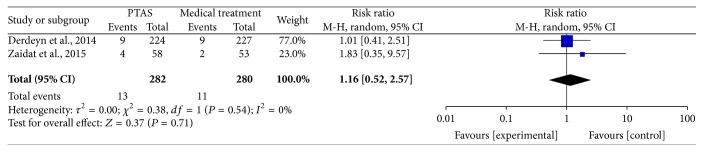
Forest plot of death within 1 year for PTAS plus medical therapy versus medical therapy alone.

**Figure 17:**
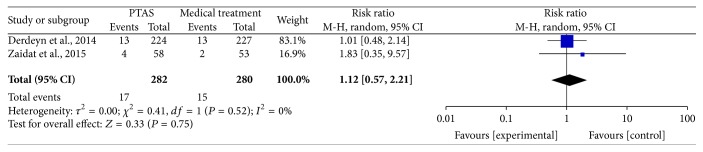
Forest plot of death during follow-up for PTAS plus medical therapy versus medical therapy alone.

The SAMMPRIS and VISSIT trials reported any death within 30 days, beyond 30 days, between 30 days and within 1 year, within 1 year, and during the follow-up. Comparing PTAS plus medical therapy with medical therapy alone, there was no heterogeneity from any death within 30 days (*P* = 0.51; *I*^2^ = 0%), beyond 30 days (*P* = 0.55; *I*^2^ = 0%), between 30 days and 1 year (*P* = 0.61; *I*^2^ = 0%), within 1 year (*P* = 0.54; *I*^2^ = 0%), and during the follow-up (*P* = 0.52; *I*^2^ = 0%). The pooled results showed no significant differences in any death within 30 days (RR = 1.14; 95% CI: 0.43–3.00; Figure 13), beyond 30 days (RR = 0.88; 95% CI: 0.32–2.41; Figure 14), between 30 days and 1 year (RR = 0.73; 95% CI: 0.16–3.31; Figure 15), within 1 year (RR = 1.16; 95% CI: 0.52–2.57; Figure 16), and during the follow-up (RR = 1.12; 95% CI: 0.57–2.21; Figure 17).

### 4.6. Myocardial Infarction during Follow-Up

**Figure 18:**
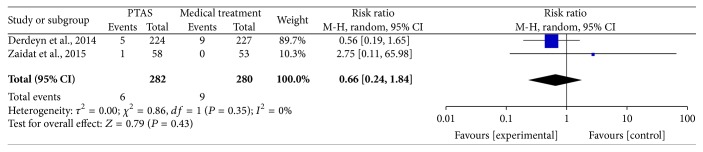
Forest plot of myocardial infarction during follow-up for PTAS plus medical therapy versus medical therapy alone.

The SAMMPRIS and VISSIT trials reported the myocardial infarction during follow-up. Comparing PTAS plus medical therapy with medical therapy alone, there was no heterogeneity from the myocardial infarction during follow-up (*P* = 0.35; *I*^2^ = 0%). The pooled results showed no significant difference in myocardial infarction during follow-up (RR = 0.66; 95% CI: 0.24–1.84; Figure 18).

## 5. Discussion

This meta-analysis indicated that any stroke, ischemic stroke, and intracranial hemorrhage within 30 days in medical therapy alone were lower, compared with PTAS plus medical therapy. But there were no significant differences in any stroke and ischemic stroke beyond 30 days between two groups. This indicated that stroke in PTAS plus medical therapy occurred in early period after operation. The SAMMPRIS trial was the first randomized trial to compare PTAS plus medical therapy with medical therapy alone. This trial enrolled 451 patients who had a TIA or nondisabling stroke within 30 days attributed to angiographically verified stenosis of 70 to 99% of the diameter of a major intracranial artery at 50 sites in the United States, and PTAS was performed under general anesthesia with the Gateway PTA Balloon Catheter and Wingspan Stent System [10, 13]. The high 30-day rate of stroke or death in PTAS group was the main reason for bad outcomes in the PTAS group, and 75% (25/33) of the events occurred within 24 hours of stenting [16] implying the flaws in study design (such as the patient selection). Dramatically, beyond 30 days the rate of stroke or death was not significantly different between the two groups [13], similar to the results of our meta-analysis, which meant that the PTAS was safe for long time follow-up. Similarly since we could not obtain the results of intracranial hemorrhage beyond 30 days, we still concluded the intracranial hemorrhage occurred in early period after operation according to the results of intracranial hemorrhage within 30 days and during follow-up.

Patients in the VISSIT trial had symptomatic intracranial stenosis (70%–99%) involving internal carotid, middle cerebral, intracranial vertebral, or basilar arteries and had a transient ischemic attack (TIA) or nondisabling stroke attributable to the territory of the target lesion within the past 30 days, and this trial was terminated due to the low likelihood of detecting superiority of stenting over medical therapy after 112 patients were randomized under the current trial design. The VISSIT trial was different from the SAMMPRIS trial in the type of stent (PHAROS™ Vitesse balloon-expandable neurovascular stent in VISSIT trial) but yielded similar outcomes to the SAMMPRIS trial [14], which indicated that the type of stent might not be related to the complications; and recent studies also suggested that the complication rates of balloon-expandable stents were similar to those of self-expanding stents [17–19]. The VAST enrolled patients who had vertebrobasilar TIA or minor ischemic stroke in the previous 6 months and had vertebral artery stenosis of at least 50% in the Netherlands and this meta-analysis only selected patients with intracranial vertebral artery stenosis in this trial. VAST was stopped for some reasons and only 16 patients with intracranial vertebral artery stenosis were randomized. The 22% of patients (2/9 patients) with intracranial vertebral artery stenosis in the stenting group had a periprocedural vertebrobasilar stroke, which was the worst result in the three studies [15].

Meanwhile, any stroke, ischemic stroke, and intracranial hemorrhage during follow-up in medical therapy alone were lower, compared with PTAS plus medical therapy and the rate of periprocedural stroke after PTAS was higher than expected. The reasons could be as follows: the medical therapy has changed during the period of the above three studies, dual antiplatelet therapy became more common, the control of low density lipoprotein cholesterol and blood pressure became more strict, the intervention of life style became more important, and treatment began more timely. However, we could find that periprocedural complication was the main reason for the bad outcomes in PTAS group, and the lower the rate of periprocedural complication, the better the outcomes.

Recently several studies showed a lower rate of periprocedural complication in PTAS plus medical therapy for ICAD. Jiang et al. reviewed 637 patients with symptomatic ICAD at 5 high-volume centers (4 in the United States and 1 in China). The overall 30-day periprocedural complication rate was 6.1% [18]. Miao et al. recruited 158 patients with symptomatic ICAD caused by hypoperfusion combined with poor collateral flow and used tailored angioplasty and/or stenting. The 30-day rate of composite stroke, myocardial infarction, or death was 4.4% (7/158) [20]. Li et al. reviewed 433 consecutive patients with intracranial arteries stenosis ≥ 70% and with symptomatic ischemic stroke or TIA (over 24 hours from the final TIA event and over 7 days from the final stroke) who underwent intracranial Wingspan stenting, and 30-day stroke rate was 6.7% (29/433) [21]. Miao et al. enrolled patients with TIA or stroke within the past 90 days due to hypoperfusion in the territory of the target ICAD and excluded patients with acute infarcts within 3 weeks. Tailored endovascular treatment of using balloon-mounted stent or balloon plus self-expanding stent for ICAD was based on anatomical features and lesion morphology. The 30-day rate of stroke, TIA, and death was 4.3% (13/300) [22]. Gao et al. enrolled patients with recent TIA or ischemic stroke related to high-grade stenosis of a major intracranial artery and with distal hypoperfusion and/or cortical involvement but excluded patients who had ischemic symptoms within the recent 3 weeks and perforator ischemic events. As a result, the overall 1-month stroke and/or death rate was 2% (2/100) [23]. Characteristics of the above trails were summarized in Table 2.

The low complication of the above studies might be related to the following reasons: investigators' experiences, patient selection, vascular morphology (lesions length, target vessel diameter, or vessel tortuosity, plague positive and negative remodeling, and problem of perforator vessel), and the pathogenesis of ischemic stroke (perfusion deficits and longer time interval before the PTAS imply a stable plaque and may reduce the risk and complications [24, 25]). We thought that the pros and cons of these methods require a clear decision-making based on individual features. Effective exploration for PTAS is on the way forever, and the design of the studies should take imaging techniques, lesion features, stent type, different stages of ischemic stroke, and procedural techniques into consideration [26, 27]. There are many different endovascular treatments such as angioplasty plus low radial force self-expanding stent, balloon-expandable drug-eluting stent, high radial force self-expanding stent without angioplasty, and drug-eluting balloon with low or high radial force self-expanding stent. The improvement of stent might also provide the changes in efficacy. Undersized balloon angioplasty and deployment of an enterprise stent with reduced radial force have been proved safe and effete for intracranial stenosis [28, 29]. Neuroimaging is an important tool in clinical trials [30]. In recent years, there are more and more new techniques to help in directing the stent placement and secondary stroke prevention [31]. High-resolution magnetic resonance imaging (HRMRI) could display the features of arterial wall, which may be useful in identifying high-risk lesions for PTAS and selecting patients for intracranial PTAS [32–35].

In addition, the role and effect of PTAS may vary according to the different phases of ischemic stroke. In 2015, five RCTs have proved the efficacy of endovascular thrombectomy by using stentriever over standard medical care in patients with acute ischemic stroke caused by occlusion of arteries of the proximal anterior circulation, so endovascular thrombectomy has been recommend as the first-line method in recanalization therapy for large artery occlusion of acute anterior circulation [36–40]. Extending the time window of endovascular thrombectomy and improving reperfusion were important in acute ischemic stroke [41–43]. Based on these therapies, some physicians considered that PTAS can be used as a rescue treatment for failure of mechanical thrombectomy for large artery occlusion of anterior circulation [44].

The present meta-analysis still has some limitations. Only 3 eligible RCTs with 581 participants were included in this meta-analysis and the sample size is inadequate. In VAST with only 19 patients involved in meta-analysis for any stroke within 30 days, the publication bias might exist. Moreover, although this meta-analysis had put equal emphasis on publications during literature search, there may be unpublished data beyond our search.

## 6. Conclusion

This meta-analysis demonstrated that any stroke and ischemic stroke in PTAS plus medical therapy occurred in early period after operation, and beyond 30 days the PTAS was not inferior compared with medical therapy alone. Periprocedural complication was the main reason for the bad outcomes in PTAS group, and the lower the rate of periprocedural complication, the better the outcomes. To reduce the rate of periprocedural complication, design of further studies should take imaging techniques, lesion features, stent type, different stages of ischemic stroke, and procedural techniques into consideration.

## Figures and Tables

**Table 1:** Characteristics of the RCTs, comparing PTAS plus medical therapy with medical therapy alone.

	Participants	Follow-up	Eligibility criteria	Stenosis rate	Stenosis location	Primary end point
SAMMPRIS	451	32.4 months	TIA or nondisabling stroke within 30 days	70%–99%	Major intracranial arteries	Any stroke or death, myocardial infarction, and any major hemorrhage

VISSIT	111	1 year	Hard TIA or stroke within the past 30 days	70%–99%	Intracranial internal carotid, middle cerebral, intracranial vertebral, or basilar arteries	Any stroke or death, hard TIA, NIHSS, and mRS scores

VAST	19^†^	3 years	Vertebrobasilar TIA or minor ischemic stroke in the previous 6 months	≥50%	Intracranial vertebral arteries	Vascular death, myocardial infarction, or any stroke

^†^The VAST included 115 patients but only 19 of them were located in intracranial vertebral artery; SAMMPRIS: Stenting and Aggressive Medical Management for Preventing Recurrent stroke in Intracranial Stenosis; VISSIT: Vitesse Intracranial Stent Study for Ischemic Stroke Therapy. VAST: Vertebral Artery Stenting Trial; TIA: transient ischemic attack; NIHSS: National Institute of Health Stroke Severity Scale; mRS: modified Rankin Scale.

**Table 2:** Characteristics of the trails mentioned in the Discussion.

Author/year	Cases	Trail detail	Eligible patients	Stenosis (% mean ± SD)	Stenosis location	Stent type	Any stroke and death at 30 days (%)
Jiang et al./2012	637	Multicenter retrospective study of consecutive patients	Symptomatic ICAD	78 ± 12	Intracranial ICA, MCA, BA, intradural VA	BES or SES	6.1

Miao et al./2015	158	Single center prospective cohort study	Symptomatic ICAD caused by hypoperfusion combined with poor collateral flow	82.01 ± 7.43	Intracranial ICA, MCA, BA, intradural VA	BES for smooth access and Mori A lesion, SES for tortuous access and Mori B or C lesion, and angioplasty alone for tortuous access and Mori A lesion	4.4

Li et al./2015	433	Single center prospective study of consecutive patients	Over 24 hours from the final TIA event and over 7 days from the final stroke caused by ICAD	82.3 ± 7.6	Intracranial ICA, MCA, BA, intradural VA	SES	6.7

Miao et al./2015	300	Multicenter prospective single-arm registry study	Symptomatic ICAD combined with poor collaterals and acute infarcts within 3 weeks were excluded	84.3 ± 7.51	Intracranial ICA, MCA, BA, intradural VA	BES or SES	4.3

Gao et al./2016	100	Multicenter prospective single-arm trial	TIA or ischemic stroke caused by ICAD and ischemic symptoms within 3 weeks were excluded	82.7 ± 8.9	Intracranial ICA, MCA, BA, intradural VA	SES	2

SD, standard deviation; ICAD, intracranial atherosclerotic disease; MCA, middle cerebral artery; VA, vertebral artery; ICA, internal carotid artery; BA, basilar artery BES, balloon-expandable stent; SES: self-expanding stent; TIA transient ischemic attack.
